# Augmentation and repair of tendons using demineralised cortical bone

**DOI:** 10.1186/s12891-016-1323-1

**Published:** 2016-11-17

**Authors:** Sherif Elnikety, Catherine J. Pendegrass, Roberta Ferro de Godoy, Charles Holden, Gordon W. Blunn

**Affiliations:** John Scales Centre for Biomedical Engineering, Institute of Orthopaedics and Musculo-Skeletal Science, University College London, Brockley Hill, Stanmore, Middlesex, HA7 4LP United Kingdom

**Keywords:** Tendon, Ligament, Demineralised bone, Scaffold, Tissue engineering

## Abstract

**Background:**

In severe injuries with loss of tendon substance a tendon graft or a synthetic substitute is usually used to restore functional length. This is usually associated with donor site morbidity, host tissue reactions and lack of remodelling of the synthetic substitutes, which may result in suboptimal outcome. A biocompatible graft with mechanical and structural properties that replicate those of normal tendon and ligament has so far not been identified. The use of demineralised bone for tendon reattachment onto bone has been shown to be effective in promoting the regeneration of a normal enthesis. Because of its properties, we proposed that Demineralised Cortical Bone (DCB) could be used in repair of a large tendon defect.

**Methods:**

Allogenic DCB grafts in strip form were prepared from sheep cortical bone by acid decalcification and used to replace the enthesis and distal 1 cm of the ovine patellar tendon adjacent to the tibial tuberosity. In 6 animals the DCB strip was used to bridge the gap between the resected end of the tendon and was attached with bone anchors. Force plate analysis was done for each animal preoperatively and at weeks 3, 9, and 12 post operatively. At week 12, after euthanasia x-rays were taken and range of movements were recorded for hind limbs of each animal. Patella, patellar tendon – DCB and proximal tibia were harvested as a block and pQCT scan was done prior to histological analysis.

**Results:**

Over time functional weight bearing significantly increased from 44% at 3 weeks post surgery to 79% at week 12. On retrieval none of the specimens showed any evidence of ossification of the DCB. Histological analysis proved formation of neo-enthesis with presence of fibrocartilage and mineralised fibrocartilage in all the specimens. DCB grafts contained host cells and showed evidence of vascularisation. Remodelling of the collagen leading to ligamentisation of the DCB was proved by the presence of crimp in the DCB graft on polarized microscopy.

**Conclusion:**

Combined with the appropriate surgical techniques, DCB can be used to achieve early mobilization and regeneration of a tendon defect which may be applicable to the repair of chronic rotator cuff injury in humans.

## Background

Tendon injuries affect millions of people every year [1] with the pattern dependent traumatic tendon injuries are more common in athletes and young adults, whereas, degenerative injuries are more common in the elderly.

Tempelhof et al. [2] found that 51% of people above 80 years of age had full thickness rotator cuff tears. Achilles tendon injury, which is the second most common form of tendon rupture, is estimated to affect one in every 10 persons below the age of 45 years [3] and is a traumatic failure associated with tendon degeneration.

Treatment of tendon injury depends on many factors, including the mechanism of injury, type of injury, patient fitness and the severity of the tendon damage. The most widely used treatment options are anti-inflammatory medications, physiotherapy and surgical treatment. In Achilles tendon rupture, a period of immobilization using a cast, followed by gradual dorsiflexion is common treatment. In complete tendon rupture, acute surgical repair is usually the option of choice for restoring function, avoiding muscle atrophy and preventing degenerative changes in the surrounding structures [4].

Complete tendon rupture resulting from direct trauma, indirect trauma (typically in athletes with acceleration/deceleration injury) or as a progression of degenerative or inflammatory changes in the tendon is a challenging condition to treat [5, 6]. Trauma might be associated with loss of tendon substance, which necessitates the use of a tendon graft to restore length. Inflammatory and degenerative changes in the tendon might indicate poor tendon quality and predisposes to the failure of surgical repair.

The duration of the tendon rupture prior to the repair affects the surgical treatment decision. Chronic tendon rupture results in tendon stiffness, tendon retraction, muscle atrophy and loss of function of the related joint. In chronic tendon rupture, direct repair might not be possible and a tendon graft is usually needed to restore length and ensure adequate repair [7].

Many tissue engineers are investigating different types of synthetic and biological tendon grafts [8]. Biological grafts rely on their ability to integrate with host tissue, which involves resorption, remodelling and replacement of the graft by the host tissues. However, autografts may illicit donor site morbidity, whilst allografts and xenografts may induce an immunological reaction, risk transferring pathogens, have problems with initial strength and are subject to different preparation schedules which may affect their performance. Advances in synthetic tendon grafts involve enrichment with cellular seeding and cytokines [9]. The goal of using biological grafts is to provide a scaffold that ideally has adequate initial mechanical strength, does not trigger an immune reaction and allows host cells to remodel its structure. Tissue engineering approaches have incorporated tendon graft scaffolds with MSCs or tenocytes, which may be complemented by growth factors and this may result in enhanced tenogenesis and better integration of the graft in the future [8, 10].

Tendon ruptures usually occur at the tendon bone interface rather than mid-substance, affecting the tendon enthesis. Repair of the tendon usually results in formation of an indirect enthesis with a scar-like tissue. Indirect enthesis may result in suboptimal outcome and loss of function. Research in tissue engineering is trying to produce biologically active grafts that provide adequate scaffold strength with no immunological reaction and remodelling potential. These grafts are expected to allow host tissue invasion and act as a carrier for growth factors that modulate the production of the direct enthesis.

Previously, research [11] has shown that the use of Demineralised Cortical Bone (DCB) for tendon repair at the enthesis results in the development of a functional interface with the formation of calcified and non-calcified cartilage linked into the bone and tendon with ‘Sharpey’s-like’ collagen fibres. This study used a thin piece of DCB sandwiched between the bone and the tendon end. Demineralised bone was first described by Urist in 1965 [12] to be osteoinductive. Reddi and Anderson found that demineralised bone results in bone induction through endochondral ossification, which is the formation of cartilage that later turns into bone by the process of mineralization of the extracellular matrix [13]. It is thought that the osteoinductive ability of the demineralised bone is mediated by BMPs and other growth factors contained in the matrix [14, 15]. Following on, the isolation of active proteins from demineralised bone eventually led to the discovery of BMPs.

Collagen of the demineralised bone is mainly collagen I, mostly oriented in longitudinal fashion following Haversian system orientation and can be processed from autogenic, allogenic and xenogenic sources. Although the collagen scaffold of the demineralised bone presents a promising material for tendon tissue engineering, very few studies have investigated its potential.

The effect of the growth factors available in demineralised bone on tendon tissue is not yet entirely clear. Evidence from literature suggests that different BMPs found in demineralised bone result in differentiation of the MSCs into osteogenic, chondrogenic and tenogenic lineage [16, 17]. Rodeo and colleagues investigated the role of BMP-2 in tendon bone healing and concluded that BMP-2 enhances this healing and results in stronger tendon-bone attachment [18–20]. Similar findings were also found by other research groups [21–24]. BMP-2 and BMP-7 also affect collagen I expression and production by human tenocytes derived from the rotator cuff [25]. BMP-2 and BMP- 4 were found to have a positive effect on osteocalcin and ALP expression by MSCs as an indication of osteoblastic activities [26–28]. Moreover, increased ALP and collagen production were found to be due to a direct effect of BMP-2 on osteoblasts [29]. Asahina et al. [30] found that BMP-7 induced chondrogenic differentiation of murine clonal cells.

Demineralised bone therefore has strong potential for being a tendon graft material; the strongly orientated collagen scaffold contains variable growth factors that are thought to enhance tendon healing and regeneration. The aim of this study is to determine whether demineralised cortical bone can be used in repair and augmentation of a large tendon defect at the enthesis. This study tests the hypothesis that demineralised cortical bone implanted in tendon environment can be remodelled into tendon, producing a direct enthesis and providing functional and competent repair. To test this hypothesis DCB was used as tendon graft to replace patellar tendon defect in live sheep. DCB-patellar tendon construct was harvested after 12 weeks and functional, radiological and histological analyses were done.

## Methods

Six skeletally mature female Friesland ewes, 2 to 3 years old, weighing between 78 and 97 kg were selected for this study. All animals were owned by the Royal Veterinary College (Hawkshead Lane, Hatfield, Hertfordshire, AL9 7TA, United Kingdom) and were allocated for research purposes. All 6 animals underwent surgical excision of the distal 1 cm of the right hind limb patellar tendon; the defect was repaired using a strip of demineralised cortical bone. The left hind limbs of the same animals were used as controls; animals were freely mobilized in individual pens and specimens were retrieved after 12 weeks. Force plate analysis, X-ray radiographs, pQCT scans and histological analysis were performed.

### DCB manufacture

DCB was manufactured according to the Urist technique [12] with some modifications. Tibias of skeletally mature female ewes aged between two and three years were harvested immediately post euthanasia; all soft tissues and periosteum were stripped from the bone surface. Proximal and distal ends of each tibia were excised and the shafts were cut into three longitudinal strips corresponding to the three surfaces of the triangular shape of the tibia using a diamond edged band saw (Exackt, Hamburg, Germany). Each strip was 3-4 mm in thickness, 17 mm (+/- 2 mm) wide and average length was 18 cm. Strips were then demineralised in 0.6 N hydrochloric acid (HCL) at room temperature. The solution was changed every 8 to 12 h until complete demineralization was achieved. Each strip was demineralised in 1 L of the HCL solution with an average two to three changes. Demineralization was confirmed by taking radiographs (300 s, 30 kV, Faxitron Corporation, Illinois, USA). Each strip was washed with phosphate buffered saline (PBS) several times until pH was around (7.4 +/- 0.1).

### Surgical procedure

**Fig. 1:**
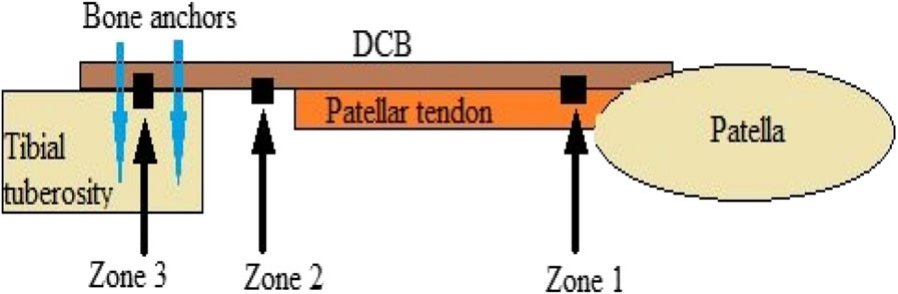
Graphic representation of the repaired tendon with the three zones examined. Zone 1: DCB-tendon interface, Zone 2: DCB alone in the region of the tendon defect. Zone 3: DCB neo-enthesis

An ovine model similar to the one developed by Sunder et al. [11] was used but 1 cm of the tendon adjacent to the tibial tuberosity was removed and the defect augmented with DCB strip which was attached to the osteotomised tibial tuberosity (Fig. 1). Surgery on 6 mature female ewes was carried out in compliance with the UK’s Animals Scientific Procedures Act. Animals were fasted overnight and pre-operative sedation was given using 0.1 mg/kg of Xylazine 2% (Bayer Health Care, Newbury, Berkshire). Anaesthesia was induced by intravenous combination of 2.5 mg Midazolam (Hypnovel, Roche Products Limited, Welwyn Garden City, UK) and 2 mg/kg Ketamine (Ketaset, Fort Doge Animal Health Ltd, Southampton, UK). Anaesthesia was maintained with 2% Isoflurane (Abbott Laboratories Ltd, Maidenhead, Berkshire, UK) mixed with pure oxygen via an endotracheal tube.

Perioperative antibiotic prophylaxis was given in the form of 2 g of intramuscular Cefalexin (Ceporex®, GSK, Brentford, Middlesex, UK) preoperatively and 1 g intramuscularly every 12 h post-operatively for three days.

The surgical field was prepared by shaving the area around the right stifle joint and decontaminating with Povidone-iodine antiseptic surgical scrub (Videne, Ecolab Inc., St. Paul, MN, USA) and Chlorhexidine surgical scrub (Hydrex, Ecolab Inc., St. Paul, MN, USA).

**Fig. 2:**
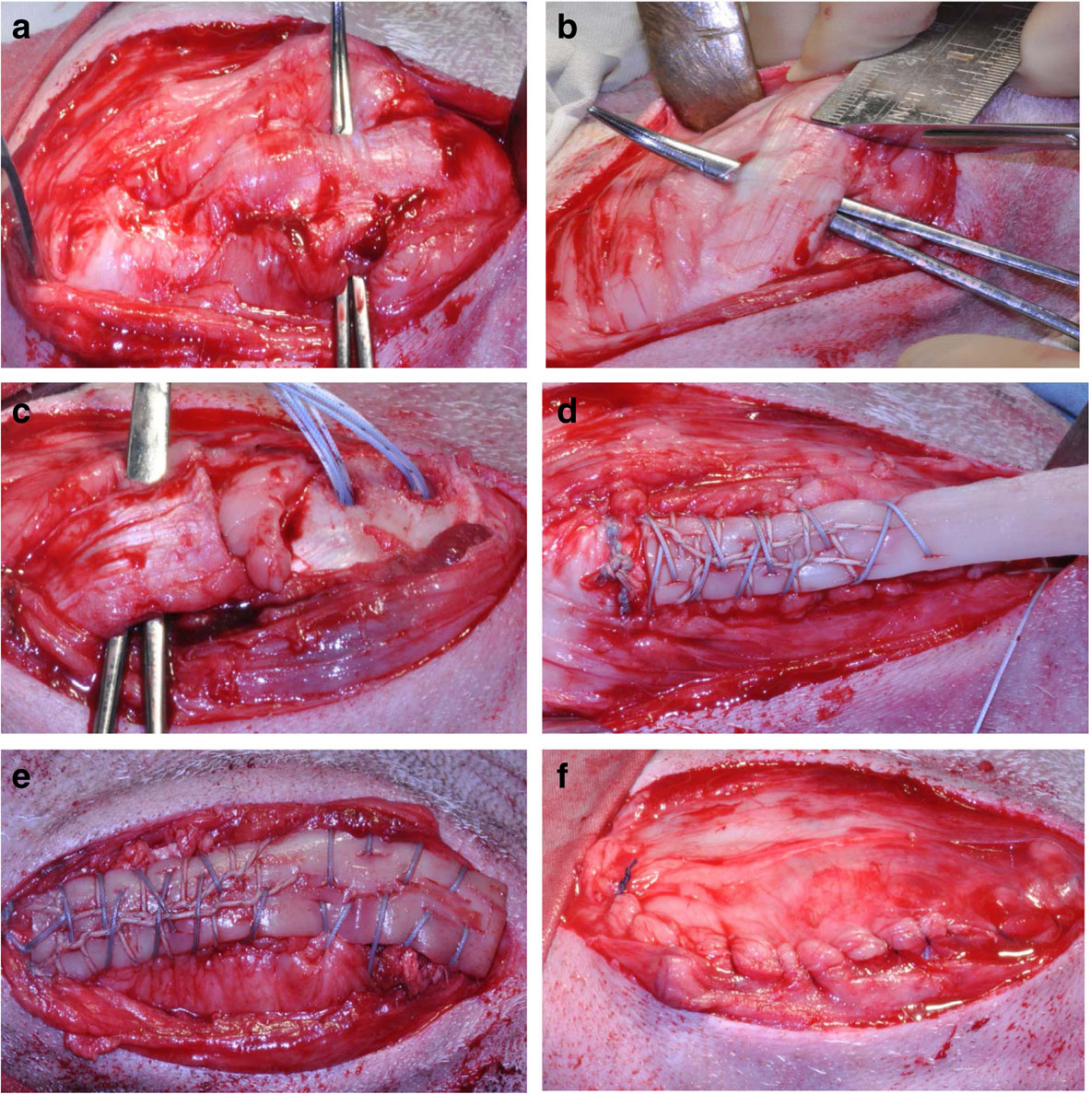
Photos showing the surgical technique: **a** Identification of patellar tendon. **b** Distal 1 cm resected. **c** Tibial tuberosity osteotomised and two suture bone anchors inserted. **d** DCB measured to match the size of the patellar tendon. **e** DCB stitched in position, **f** Closure in layers

A longitudinal skin incision was made extending from above the patella distal to the tibial tuberosity and was made over the right stifle joint. The proximal end of the patellar tendon insertion into the tibial tuberosity was identified. One cm of the patellar tendon proximal to this was surgically incised and the distal insertion into the tibial tuberosity was surgically avulsed by sharp dissection. Complete surgical avulsion of the patellar tendon insertion was confirmed by retraction of the patellar tendon away from the tibial tuberosity. The footprint of the insertion of the patellar tendon on the tibial tuberosity was osteotomised and two holes were drilled 1 cm apart on the prepared flat bone surface (Fig. 2). The size of the DCB strip was adjusted to match the length and the width of the patellar tendon prior to tendon repair.

Two bone suture anchors (Corkscrew, Arthrex, Naples, Florida, USA) were used to stitch the DCB to the patellar tendon. Anchors were inserted in the drilled holes and sutures from both anchors were used to stitch the DCB to the patellar tendon using the whipstitch technique. The distal end of the DCB strip overlying the tibial tuberosity was also stitched to the surrounding tissue to ensure direct contact with the bony surface. Closure of the surgical wound was done in layers using absorbable Vicryl sutures (Ethicon Inc., Somerville, NJ, USA).

### Force plate analysis

Kistler force plate analysis was conducted to determine the functional weight bearing (FWB) status of the left and right hind limb in each animal. The assessments were carried out pre-operatively and at weeks 3, 6, 9, and 12. All measurements were performed in a Gait Analysis Laboratory by walking animals over a force plate (Kistler Biomechanics Limited, Alton, UK). For each animal in the study, the 12 readings of the vertical components of the ground reaction force (GRFz) for both the left and right hind limbs were recorded. Excessively high or low readings (10 standard deviations outside the average) were rejected. Readings were also discarded if more than one limb was in contact with the force plate or contact was for more than 900 ms. The mean GRFz was calculated (Fmax) and normalized against the animals' weight (Fmax/weight) at the time of the study. The FWB was calculated as the mean GRFz of the right hind limb (operated limb) over the mean GRFz of the left hind limb (non-operated limb) expressed as a percentage.

### Post-operative care

After recovery, animals were moved to individual pens of average size 2 m X 2 m. There was no weight-bearing protection and animals were allowed to freely mobilise immediately post-operatively. No limitations were applied on range of movement; the operated limb was freely mobile as pain allowed. Post-operative analgesia was maintained by Fentanyl transdermal patches (Duragesic®, Janssen Pharmaceuticals, NJ, USA), the first one 12 h post-operatively and the second one 60 h after the first patch. At weeks 3, 6, 9 and 12, animals were moved to the gait analysis laboratory for the force plate analysis, as previously described.

### Euthanasia and retrieval of the specimens

At week 12, after the force plate analysis, animals were given an overdose of 0.7 mg/kg of 20% Pentobarbital (J. M. Loveridge, Southampton, UK). Digital lateral X-ray radiographs of both hind limbs were taken in full flexion and full extension (Raymax, Elstree, Middlesex, UK). Flexion and extension angles of each digital radiograph were measured using Image J software (ImagJ 1.48 g, National Institutes of Health, USA). Two lines parallel to the longitudinal axes of both femur and tibia in each radiograph were drawn and the angle at the transaction of these two lines was measured and recorded.

Immediate retrieval with dissection of the patella, patellar tendon construct and proximal tibia all in block was done for both hind limbs of each animal.

Morphological assessment of the construct and the surrounding tissues was documented during the dissection. Then, a pQCT scan (XCT2000, Norland Stratec, Norland, Wisconsin, USA) was done to look for peripheral ossification prior to fixation in a 10% formaldehyde solution.

### Sample processing and sections production

Samples were fixed in a 10% formaldehyde solution before dehydration using ascending concentrations of 50%, 75%, 85%, 95% and 100% of industrial Methylated spirit. Samples were then treated with 100% chloroform, further processed into 100% IMS before infiltrating and being embedded with methyl methacrylate resin (LR White Hard Grade Resin, London Resin Company Limited, Reading, UK). Blocks of hard resin containing the retrieved specimens were cut in half transversely through the middle of the patellar tendon, this construct was created for ease of handling and sections were prepared from different regions of the construct (Fig. 1).

- Zone 1: DCB-tendon interface,
- Zone 2: DCB alone in the region of the tendon defect.
- Zone 3: DCB neo-enthesis, examining the area of the new tendon enthesis over the tibial tuberosity.

Sections were mounted on acrylic slides and a precision diamond-encrusted band saw (Exakt, Apparatebau GmbH, Norderstedt, Germany) was used to cut a sections 300 μm thick. These sections were then ground and polished using the Exakt Microgrinding system to a final thickness of 60 μm. Sections stained with Toluidine Blue and Paragon for 20 min each.

### Histological examination and analysis

A qualitative histological analysis using a light microscope (Zeiss, Hamburg, Germany) with associated Axiovision image processing software was carried out. All sections were examined for evidence of ossification, inflammatory cells,

Cellularisation, vascularisation and collagen fibre crimp. Zone 1 sections were also examined for interactions between DCB and the patellar tendon, while Zone 3 sections were additionally examined for collagen fibre orientation and formation of the neo-enthesis. A semi-quantitative analysis was conducted for the neo-enthesis according to the criteria highlighted in Table 1.

**Table 1:** Criteria for the semi-quantitative analysis of the neo-enthesis

Score	Criteria
1	No fibrocartilage
	No mineralized fibrocartilage
2	Fibrocartilage present
	No mineralized fibrocartilage
3	Fibrocartilage present
	Mineralized fibrocartilage present
	Disorganized arrangement
4	Fibrocartilage present
	Mineralized fibrocartilage present
	Organized graduation between distinct regions but no tidemark
5	Fibrocartilage present
	Mineralized fibrocartilage present
	Organized graduation between distinct regions with tidemark

**Fig. 3:**
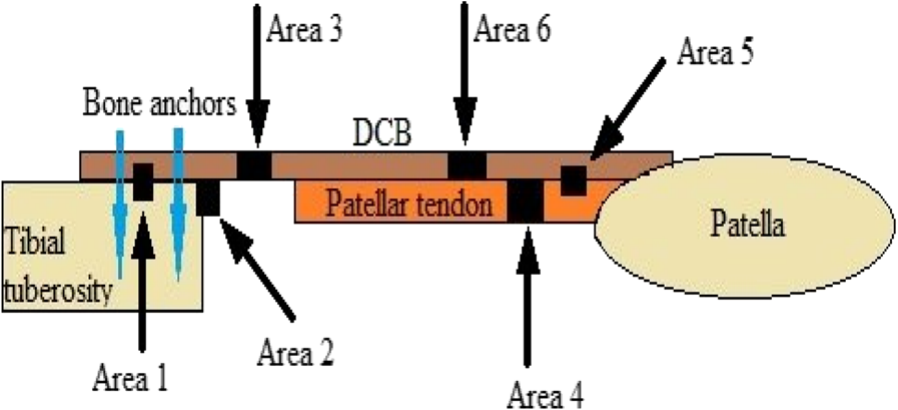
Graphic representation of different areas examined for cell counting

Three sections, taken at one third width intervals across the neo-enthesis were examined for semi-quantitative analysis by three different researchers and an average of the score for each section was taken as the final score. Results of the semi-quantitative analysis were compared with the contralateral control knees. The number of tenocytes and chondrocytes from different regions of the specimens were counted using an objective of X 40. Six distinct areas were used for cell counting (see Fig. 3).

- Area 1: Neo-enthesis, DCB above tibial tuberosity
- Area 2: New tendon bone interface
- Area 3: DCB cellularisation
- Area 4: Patellar tendon, below the tendon-DCB interface
- Area 5: Tendon-DCB interface
- Area 6: DCB above the patellar tendon.

### Statistical analysis

Statistical software package SPSS v.21 (SPSS Inc., Chicago, Illinois, USA) was used in this study; non-parametric analysis was conducted, as data did not match the requirements for parametric tests. A *p*-value of <0.05 was considered appropriate for statistical significance.

### Consent statement

This study was done on live animals, no human involvement, therefore no consent was required.

## Results

### Failure rate

**Fig. 4:**
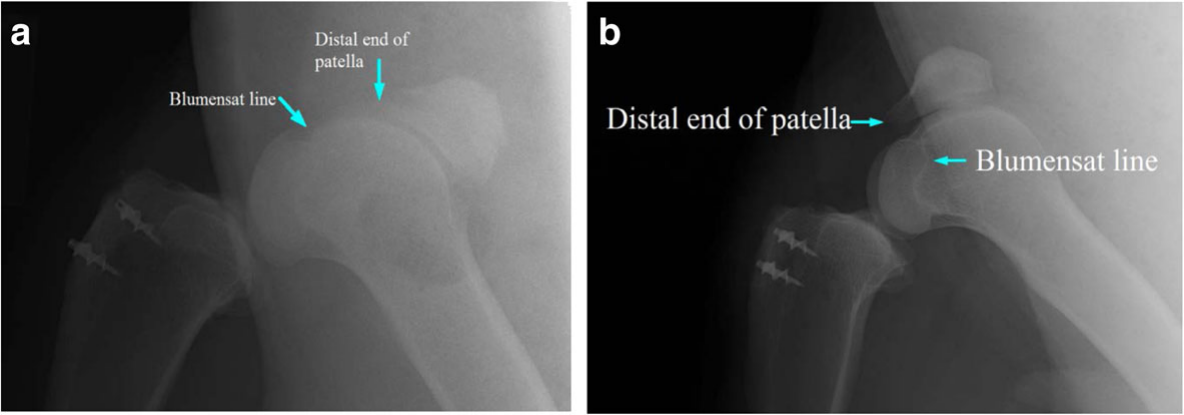
**a** lateral radiograph of the operated right knee of the failed animal showing patella alta. **b** lateral radiograph of a non-failed animal showing normally positioned patella, both at full flexion

All six animals survived the duration of the study and none had post-operative infection. Five animals showed satisfactory progression according to force plate analysis, while one animal failed to show a similar progression. Results of this animal were excluded from the force plate analysis and included in all other aspects of the study. On retrieval of specimens, the animal that failed to show satisfactory progression did not have any evidence of infection or inflammatory reaction. In this animal all suture anchors were well-positioned in the bone and suture material was intact. X-ray radiographs showed evidence of patella alta (Fig. 4). Failure of progression was explained by elongation of the patellar tendon, secondary to ‘cheesewiring’ of the suture material through the DCB and tendon substance; this might have been a result of poor surgical technique.

### Gait analysis

Obvious antalgic gait was observed post-operatively in the operated hind limb. Except for one animal that was diagnosed with patella alta, progressive improvement in the gait was observed over the study period. At 6 weeks, the antalgic gait was very mild and unrecognisable except by trained observers; at 12 weeks, all animals except for the one with patella alta had normal gait and no limping was observed.

### Force plate analysis

**Fig. 5:**
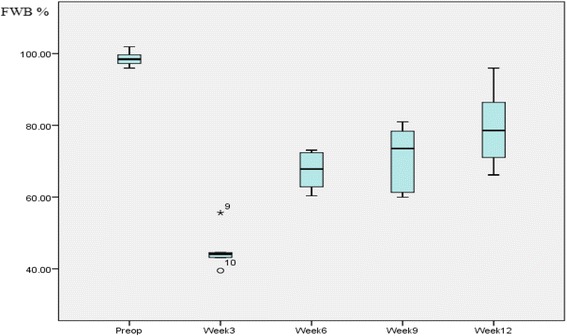
Box and whiskers plot showing progression of the FWB over time

Force plate analysis examined pre-operatively, in week 3, week 6, week 9 and week 12 showed five of the animals exhibiting satisfactory progression in FWB over time (Fig. 5).

The GRF_z_ of the right hind limb (operated limb) showed a significant drop at week 3, followed by steady recovery between week 3 and week 12. The opposite was observed for the left non-operated hind limb, that is, a mild increase in the GRFz at week 3 with steady decline over the subsequent assessment points (Table 2, Fig. 5).

**Table 2:** Median and 95% confidence intervals of right and left hind limbs GRFz at different time intervals

	Median	95% CI
Rt Preop	372.94	312.64–426.22
Lt Preop	374.22	312.14–438.04
Rt Week 3	201.87	153.7–266.38
Lt Week 3	457.84	379.86–542.16
Rt Week 6	264.84	214.71–344.8
Lt Week 6	438.64	346.3–481.79
Rt Week 9	293.53	249.31–347.88
Lt Week 9	446.96	335.37–518.62
Rt Week 12	324.42	261.65–410.62
Lt Week 12	431.36	322.04–533.27

Pre-operatively, the median FWB was 98.6% with 95% confidence interval 95.8%-101.5% (Table 4). The Mann–Whitney *U* test showed no statistical significance between the right and left hind limbs at pre-operative assessment (*p* = 0.754).

### Morphological assessment during retrieval of the specimens

**Fig. 6:**
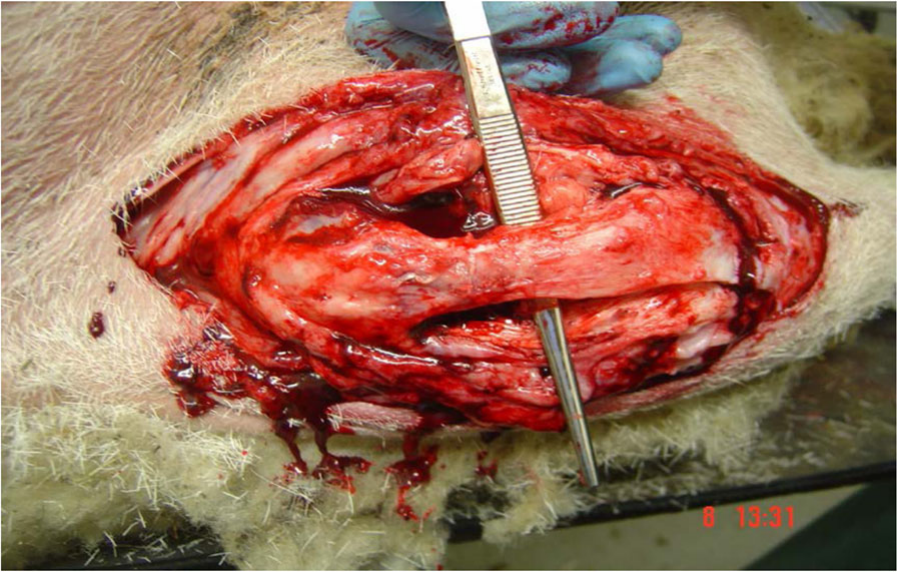
Deep tissue dissection showing well integrated DCB, showing normal tissues with no inflammation or excessive scaring

All animals showed similar findings and no evidence of superficial or deep infection was found. Normal post-operative scar tissue was present and there was no evidence of inflammatory reaction or excessive granulation tissue (Fig. 6).

Normal vasculature was also seen with no excessive hyperaemia or vasculitic lesions. Synovial fluid from the knee joint was present in normal amounts with no evidence of effusion or reactive synovitis. The DCB was well integrated into both the tibial tuberosity and the patellar tendon. The distinction between the DCB and the patellar tendon was very difficult to discern visually (Fig. 6). The suture bone anchors maintained their position in the bone with no evidence of pull-out or migration. All the stitch materials were found intact with no evidence of rupture or failure. The infrapatellar fat pad was attached to the posterior aspect of the patellar tendon, which is a normal finding as seen in the non-operated knees.

### Radiographic assessment

Lateral radiographs for both hind limbs in full flexion and full extension were taken of each animal immediately after euthanasia. No evidence of calcification of DCB was seen in any of the six animals; some bony irregularity was present at the neoenthesis over the osteotomised surface of the tibial tuberosity. The ranges of movement of the knees for both hind limbs were recorded (Table 3). The ranges of movement of the operated on limbs were comparable to those of the non-operated limbs. The Mann–Whitney *U* test did not show any statistical significance between the right and left hind limb full flexion angles (*p* = 0.065) or between the full extension angles (*p* = 0.394).

**Table 3:** Flexion and extension angles of both hind limb knees of each animal (in degrees)

	Right hind limb knee		Left hind limb knee	
	Flexion	Extension	Flexion	Extension
Animal 1	48	117	57	113
Animal 2	46	108	55	121
Animal 3	43	116	62	129
Animal 4	47	104	42	111
Animal 5	40	109	63	106
Animal 6	54	125	51	129

### PQCT scans

**Fig. 7:**
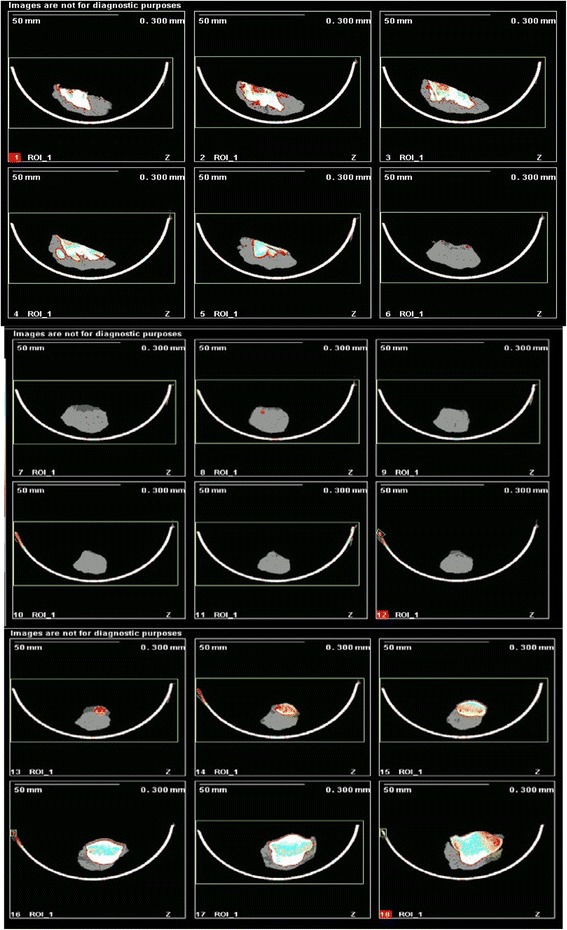
pQCT scan images showing no ossification within the patellar tendon or DCB

pQCT Scanning was performed to examine for ossification within the patellar tendon, the DCB and surrounding tissues; 5 mm sections were taken through the tibial tuberosity, patellar tendon/DCB and patella. None of the specimens showed any evidence of ossification within the DCB or the substance of the patellar tendon (Fig. 7).

### Histological analysis

#### Qualitative assessment

**Fig. 8:**
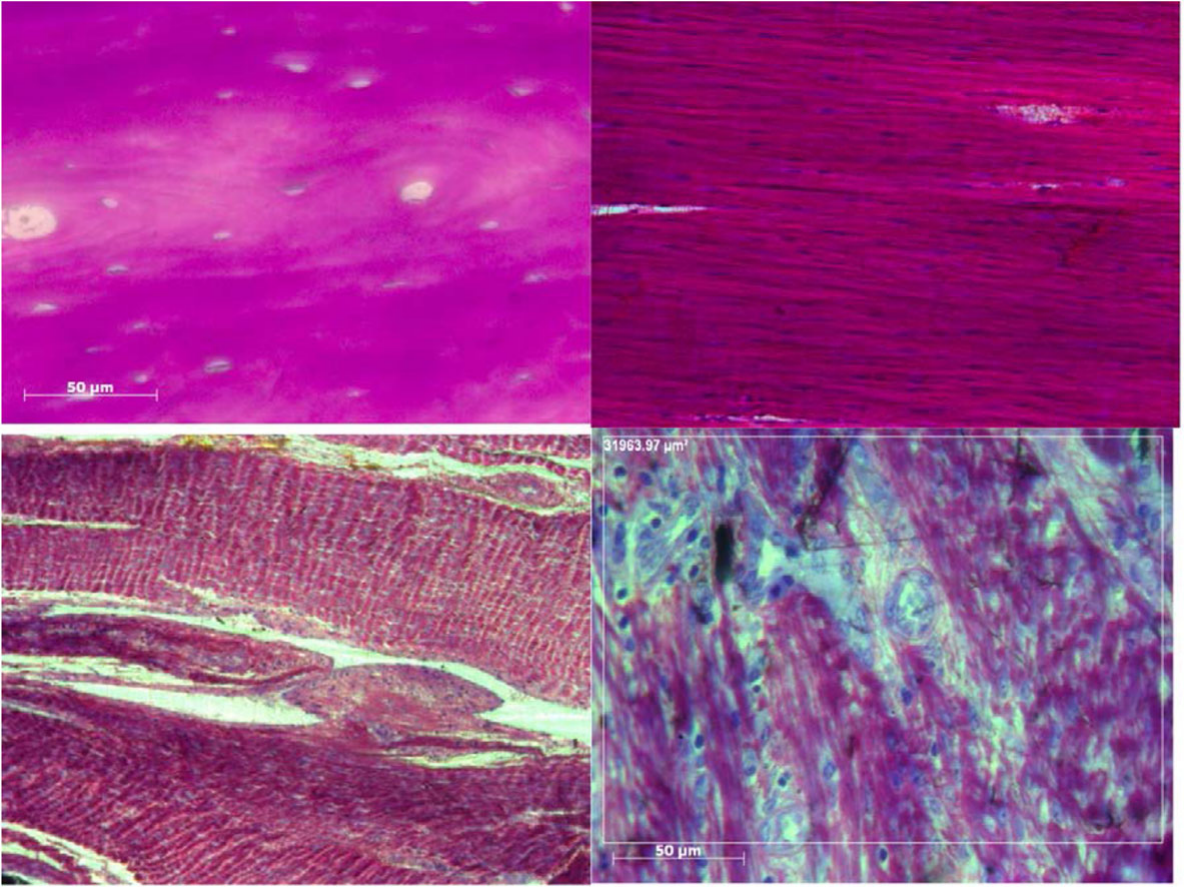
*Top left*: Histology photograph showing DCB before implantation. *Top right*: remodelled DCB §showing aligned cell infiltration. *Bottom left*: Well organised collagen fibres within DCB showing crimp. *Bottom right*: Less organised remodelling in DCB showing vascularisation and collagen fibre formation

Sections from all animals were examined (including the one that failed to recover its gait). All animals showed strong evidence of remodelling and integration. In all sections there was cellularisation (Fig. 8); tenocytes and chondrocytes were seen in all parts of the DCB and spindle shaped cells were arranged longitudinally with the oval shaped nuclei arranged in a longitudinal pattern parallel to the longitudinal axis of the patellar tendon (Fig. 8). Mature blood vessels were also found in the tissue of DCB (Fig. 8). Collagen fibres were organised in a longitudinal pattern parallel to the longitudinal access of the patellar tendon. Collagen fibres showed evidence of characteristic crimping on polarized microscopy in different areas of DCB (Fig. 8).

No changes were seen in the native patellar tendon and there was no evidence of inflammatory cells in either DCB or the patellar tendon; no lymphocytes, macrophages or other immune response cells were found in any of the sections. No evidence of resorption of DCB or heterotrophic ossification was found.

#### Zone 1: Interactions between DCB and the patellar tendon

**Fig. 9:**
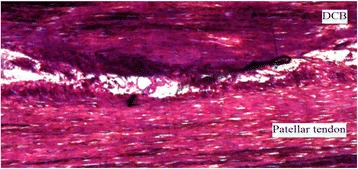
Histological photograph showing the interface between the DCB and the patellar tendon

All sections examined from all the animals showed interconnections between DCB and the native patellar tendon (Fig. 9). In some areas, DCB was well integrated into the patellar tendon with no clear demarcation between either but in others the interface could be identified (Fig. 9).

#### Zone 2: Remodelling of the DCB

Zone 2 is the region where the DCB bridges the gap between the end of the patellar tendon and the tibial tuberosity. In this zone, all the sections showed strong evidence of remodelling in the form of cellularisation, vascular invasion and crimped collagen fibres. All cells were aligned longitudinally, with oval nuclei also aligned longitudinally in line with the long access of the patellar tendon. Collagen fibres were all in longitudinal fashion; the characteristic crimp of the normal tendon was found in the absence of the Haversian system of DCB.

#### Zone 3: formation of neo-enthesis

**Fig. 10:**
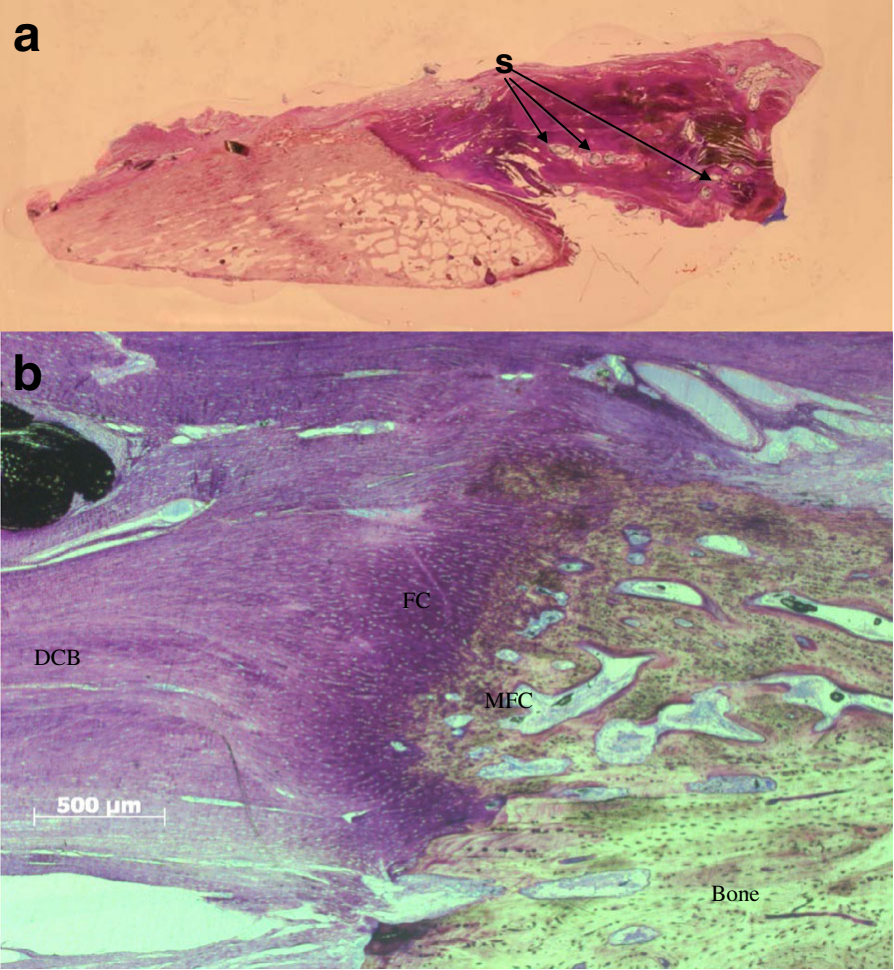
**a** Low power photograph of the enthesis showing the bone of the tibial tuberosity, the regenerative tendon attachment and sutures (S) within the DCB. **b** Higher power image of the enthesis showing bone with an irregular surface with the regenerating tendon and chondrocytes at the interface (FC Fibrocartilage, MFC: Mineralised Fibrocartilage)

Zone 3 describes the attachment of the DCB onto the osteotomised surface of the tibial tuberosity to form the neo-enthesis. In all the specimens, a direct type enthesis was found with the characteristic transition between bone, mineralized fibrocartilage, demineralised fibrocartilage and tendon (Fig. 10). Some of the sections showed a clear tidemark, differentiating between these zones with clear demarcation. Although in others no clear tidemark was seen, the four zones were observed with gradual transition between the four zones of the enthesis. Collagen fibres were also seen penetrating the fibrocartilage into the bony tissue in all sections and running parallel to the long access of the collagen fibres of DCB, which were in turn parallel to the long access of the patellar tendon and in line with the direction of tensile loading of the tendon. At the neo-enthesis, cells were arranged longitudinally and in line with the collagen fibres.

A semi-quantitative analysis of the neo-enthesis was conducted as detailed in section 3.7. This analysis aimed to examine the maturation of the neo-enthesis, the presence of the four zones and clear distinct transition between these zones. In all sections examined, the four zones of the direct enthesis were seen; there were fibrocartilage and mineralized fibrocartilage zones in all sections. In the experimental group transitions between the zones were not distinct compared with enthesis of the contralateral tibial tuberosity. The interface between the tendon and the bone in the experimental group was more irregular when compared with the enthesis of the tibial tuberosity in the contralateral tibia. The three independent observers scored enthesis in experiential group between 3 and 5 with a mean score of 4, whereas, the mean score for the natural enthesis in the contralateral limb was 4.8. There was no statistical significance between the scores of the neo-enthesis and the contralateral control enthesis on the Mann–Whitney *U* test (*p* = 0.13).

### Cellularisation of the constructs

Both chondrocytes and tenocytes were counted in different areas of the specimens. The median and confidence interval for each cell type were calculated for different areas of the specimens (Table 4). In areas one and two, where the DCB was in close proximity to the bone, the number of chondrocytes was significantly higher compared to tenocytes, whereas in the patellar tendon substance and the DCB-tendon interface (areas 4 and 5), the number of tenocytes was significantly higher. In areas 3 and 6, where the DCB was not in contact with either tendon or bone, there was no statistical significance between the number of chondrocytes and tenocytes.

**Table 4:** Chondrocyte and tenocyte cell counts in different areas of the specimens (cell/mm2)

	Chondrocytes Median (95% Confidence interval)	Tenocytes Median (95% Confidence interval)	*p*-value Chondrocytes: tenocytes
Area 1	1727 (985–2312)	377 (167–681)	0.004
Area 2	1076 (547–1489)	88 (0–319)	0.010
Area 3	608 (88–1504)	380 (66–1355)	0.520
Area 4	89 (20–209)	1014 (453–2076)	0.004
Area 5	258 (97–403)	700 (374–1138)	0.016
Area 6	1930 (419–2721)	687 (224–1205)	0.262
Control enthesis	469 (332–542)	57 (0–112)	0.004

## Discussion

We have shown that allogenic DCB in strip form can be used to replace the distal 1 cm of the ovine patellar tendon adjacent to the tibial tuberosity. This results in the formation of an enthesis, which is similar in morphology to a normal enthesis and over time functional weight bearing significantly increased from 44% at 3 weeks post-surgery to 79% at week 12. On retrieval none of the specimens showed any evidence of ossification of the DCB. Histological analysis proved formation of neoenthesis with presence of fibrocartilage and mineralised fibrocartilage in all the specimens. DCB grafts contained host cells and showed evidence of vascularisation. Remodelling of the collagen leading to ligamentisation of the DCB was shown by the presence of crimp in the DCB graft on polarized microscopy.

One animal out of six failed to demonstrate satisfactory progression on FWB status as examined on force plate analysis. This animal was excluded from the force plate study; however, it was included in all other aspects of this study. Radiological and morphological assessments of the repaired patellar tendon construct of this animal have proven the presence of patella alta (high riding patella) as demonstrated in Fig. 4. There were no other findings to explain the failure of this animal. On morphological assessment at retrieval, no difference was found between this animal and the other five animals.

In humans, the length of the patellar tendon is on average 44 mm (range 35–55 mm), according to results of cadaveric study on human knees [31]. Clinicians usually refer to the Insall-Salvati ratio [32, 33] as one of the radiological features of abnormal patellar position. Patella alta in the failed animal was based on radiological position of the distal pole of the patella in relation to the roof of the inter-condylar notch (Blumensaat line). In all five animals, the pole of the patella was distal to the horizontal line passing through at > 100 0 of flexion. In the failed animal, the distal pole was significantly proximal to this.

Whipstitch techniques in tendon repair have been widely practised in clinical repair of tendon injuries [34] and are considered superior to direct end-to-end stitching. In this study, a combination of both Krackow [35, 36] and running whipstitch techniques were used in each animal. One of the known drawbacks of the whipstitch techniques is tendon elongation and gap formation at the repair site [37]. The failure in the one animal was due to ‘cheese-wiring’ of the suture material through the DCB.

FWB and the effect of surgical intervention on weight bearing status has been established in medical and biomechanical fields [38, 39]. Previous studies have established a direct relationship between the vertical component of the ground reaction force and the forces going through the limb, which reflect on the weight-bearing status of the limb [40].

During the first three weeks of this study, the animals showed the expected postsurgical reaction of antalgic limp. During this period, the forces going through the patellar tendon were probably solely transferred through the suture material, which provided the initial mechanical strength for the repair. After 3 weeks, the animals started to recover with gradual progression into normal non-antalgic gait. While forces going through the operated on limbs were gradually increasing over time, the forces going through the non-operated limbs increased postoperatively at week 3, with gradual reduction to baseline pre-operative values. This in itself represents a clear indication of progressive healing and recovery of the operated on patellar tendon. The improvement in FWB can only be explained by the biological process of healing of the repair, as in ovine models, forces are only transmitted through the patellar tendon with no other compensatory mechanism to explain the improvement in FWB [41].

The traditional expectation of demineralised bone is that it ossifies after implantation through endochondral ossification. This is based on Urist’s work, which shows that demineralised bone contains osteoinductive cytokines and it provides a suitable scaffold for osteogenesis [42]. In his studies, Urist found that ossification of the demineralised bone implanted in soft tissue pockets proceeded by cartilage formation and endochondral ossification, was evident as early as 4 weeks. The radiographic assessment and pQCT scan results in our study showed no evidence of ossification of DCB at 12 weeks. DCB was implanted in a tendon environment that possibly released local cytokines from the patellar tendon. In addition, the mechanobiology of the construct may drive cells down a tenogenic or chondrogenic pathway and this may account for the lack of observed ossification.

As acute tendon rupture usually occurs at the enthesis, treatment aims to restore normal enthesis. Several studies have found that anatomical healing of tendon ruptures results in poorly differentiated enthesis. The healing usually starts with disorganized collagen fibres filling in the enthesis gap, which later progresses into fibrous scar-like tissue, with anchoring to the bone via increased ossification [43]. In a two-year follow-up study on the repair of enthesis in sheep, it was found that the enthesis remodels into a functional type attachment with increased organized fibrous tissue, rather than the normal four zones attachment [44]. This contrasts to our study which shows the development of a direct enthesis after a relatively short term.

Previous studies have found that using a demineralised bone matrix during the repair of tendon rupture as an interface between the tendon and bone results in improved healing, with four zone enthesis rather than fibrous enthesis [11]. This study was reinforced by this current research where a direct enthesis was identified even adjacent to a large tendon deficit.

On histological analysis of the DCB, there was strong evidence of remodelling of DCB into a ligament/tendon like structure. The Haversian system arrangement of the bony tissues was not seen, nor was the characteristic bony lacunae; instead, it was replaced with collagen fibres arranged longitudinally in line with the long axis of the tendon, with the axis of stress loading of the tendon.

The DCB overlaying the patellar tendon was found to have variable degrees of integration with the native tendon. This may be part of the remodelling process, which occurs over a long period of time, potentially up to two years, as previous studies have shown [44, 45]. Furthermore, a variable degree of crimping of the collagen fibres of DCB was seen in all the sections and this variability may be part of the remodelling process. The lack of ossification or any evidence of resorption of DCB with the presence of cellularisation, vascularisation and crimping support the remodelling process of DCB into ligament structure.

During the surgical procedure, no or very minimal dissection of the Hoffa fat pad, which is known to be a rich source of MSCs [46], was performed. These MSCs are known precursors for the tenocytes and chondrocytes needed for DCB remodelling into ligament, which was found in DCB specimens in my study [47]. Different numbers of tenocytes and chondrocytes were present in different zones of DCB and it is not clear how this was modulated.

At the neo-enthesis, a fibrocartilaginous enthesis was seen with its characteristic four-zone transition; these findings support the previous study conducted by our work group [11]. The four zones were present in all sections examined, while tidemarks between transitions were absent in some of the sections. The tidemark is not found in all areas of the normal enthesis. The presence and thickness of the tidemark varies according to the differentiation of the enthesis and the thickness of the different zones of the enthesis [48].

The limitations of this study are associated with the comparison that was made with the contralateral tendon and enthesis. Whilst the animals were recovering then greater loads were probably transferred through the intact structure of the contralateral patellar tendon. This could have led to some functional adaption. However as we have not investigated the natural tendon and enthesis in sheep with normal gait then it is difficult to say if this led to a change in the morphology. A comparison could have been made with a predicate graft material that is used commercially, however in this severe model commercially available graft material would not have been strong enough and it would have been unethical to have conducted a study on live animals knowing that a comparison at 12 weeks would not have been possible due to the failure of the commercial graft material.

## Conclusion

This study proves that DCB can be used to repair and augment tendons. The results of this research show that it remodelled into a ligament-like structure regulated by host cells. DCB produced fibrocartilaginous enthesis with its characteristic four zones. Demineralised cortical bone therefore has a strong potential for use as a biological tendon graft, which presents solutions to the current clinical limitations and restrictions concerning available grafts.

## Abbreviations

ALP: Alkaline phosphatase
BMP: Bone morphogenic protein
DCB: Demineralised cortical bone
Fmax: Mean GRFz was calculated
FWB: Functional weight bearing
GRFz: Ground reaction force
HCL: Hydrochloric acid
MSC: Mesenchymal cell
PBS: Phosphate buffered saline
pQCT: Peripheral quantitative computerised tomography
